# Confocal Laser Scanning Microscopy for Detection of *Schistosoma mansoni* Eggs in the Gut of Mice

**DOI:** 10.1371/journal.pone.0018799

**Published:** 2011-04-18

**Authors:** Martha Charlotte Holtfreter, Oliver Stachs, Maria Reichard, Micha Loebermann, Rudolf Friedrich Guthoff, Emil Christian Reisinger

**Affiliations:** 1 Division of Tropical Medicine and Infectious Diseases, Department of Internal Medicine, University of Rostock, Rostock, Germany; 2 Department of Ophthalmology, University of Rostock, Rostock, Germany; Fundação Oswaldo Cruz, Brazil

## Abstract

**Background:**

The gold standard for diagnosing *Schistosoma mansoni* infections is the detection of eggs from stool or biopsy specimens. The viability of collected eggs can be tested by the miracidium hatching procedure. Direct detection methods are often limited in patients with light or early infections, whereas serological tests and PCR methods fail to differentiate between an inactive and persistent infection and between schistosomal species. Recently, confocal laser scanning microscopy (CLSM) has been introduced as a diagnostic tool in several fields of medicine. In this study we evaluated CLSM for the detection of viable eggs of *S. mansoni* directly within the gut of infected mice.

**Methodology/Principal Findings:**

The confocal laser scanning microscope used in this study is based on the Heidelberg Retina Tomograph II scanning laser system in combination with the Rostock Cornea Module (image modality 1) or a rigid endoscope (image modality 2). Colon sections of five infected mice were examined with image modalities 1 and 2 for schistosomal eggs. Afterwards a biopsy specimen was taken from each colon section and examined by bright-field microscopy. Visualised eggs were counted and classified in terms of viability status.

**Conclusions/Significance:**

We were able to show that CLSM visualises eggs directly within the gut and permits discrimination of schistosomal species and determination of egg viability. Thus, CLSM may be a suitable non-invasive tool for the diagnosis of schistosomiasis in humans.

## Introduction

Some 200 million people suffer from schistosomiasis worldwide. Female worms residing in the mesenteric vessels shed large numbers of eggs, which penetrate through the tissues towards hollow organs such as the urinary bladder in *S. haematobium* infections and the bowel in *S. mansoni* infections. The gold standard for the diagnosis of schistosomiasis is the detection of eggs possessing a characteristic spine from urine, stool, or rectal and bladder biopsy specimens.

Direct detection of eggs is limited in patients with low egg shedding rates. Indirect immunological tests such as enzyme-linked immunosorbent assays (ELISA), immunofluorescence assays (IFA) and indirect haemagglutination assays (IHA) are widely used to detect antibodies against worm or soluble egg antigen [1]–[3]. However, these assays cannot differentiate between persistent or inactive infections and fail to discriminate between parasite species. Direct immunological tests, such as the detection of the proteoglycans circulating anodic antigen (CAA) or circulating cathodic antigen (CCA) are highly specific and correlate with egg counts, but also fail to differentiate schistosomal species [1]. While polymerase chain reaction (PCR) methods can detect schistosomal egg DNA in stool and urine or parasite DNA in serum samples even in low-intensity schistosomal infections [4]–[8], none of the published PCR methods has so far been evaluated for routine diagnosis.

The detection of viable eggs indicates an active infection requiring drug treatment. The viability of collected eggs from stool, urine and biopsy specimens can be tested by the miracidium hatching procedure, where larvae hatch from the eggs during incubation in fresh water. However, this procedure is time-consuming and has a low sensitivity [9]. The viability of the collected eggs can also be tested by labelling the eggs with the fluorescent probe Hoechst 33258. In contrast to the dead eggs, eggs morphologically classified as viable do not show fluorescence [10].

Confocal laser scanning microscopy (CLSM) allows non-invasive cell imaging *in vivo* and has been established for obtaining high-resolution images and 3-dimensional reconstructions [11]. For microscopy the light source, usually a laser, is focused at a point within the tissue. For imaging this focal point can be scanned in the x- and y- directions. The light emitted back from the tissue is separated from the excitation light and the focal point is imaged onto a pinhole in front of the detection system. Evaluating changes in the refractive index permits recognition of intercellular and intracellular details and tissue alterations. CLSM has recently been introduced as a diagnostic tool in ophthalmology, urology, dermatology, gastroenterology and oncology [12]–[16].

The aim of the present study was to evaluate CLSM for visualisation of viable eggs of *S. mansoni* directly within the gut of infected mice.

## Materials and Methods

### Imaging

The CLSM device (wavelength 670 nm, image size 348×384 pixels, 30 frames per second) used in this study was based on a scanning laser system for imaging the retina of a living eye known as the Heidelberg Retina Tomograph II (HRT II, Heidelberg Engineering GmbH, Germany) in combination with two different lens systems (image modalities 1 and 2). Both lens systems covered a field of view of 400×400 µm and were coupled to the mucosal tissue with interposition of transparent gel (Vidisic, Mann Pharma, Germany). The penetration depth of both optical systems is about 100 µm and is limited by signal-to-noise ratio and background intensity. Pinhole size was fixed.

### Image modality 1

**Figure 1:**
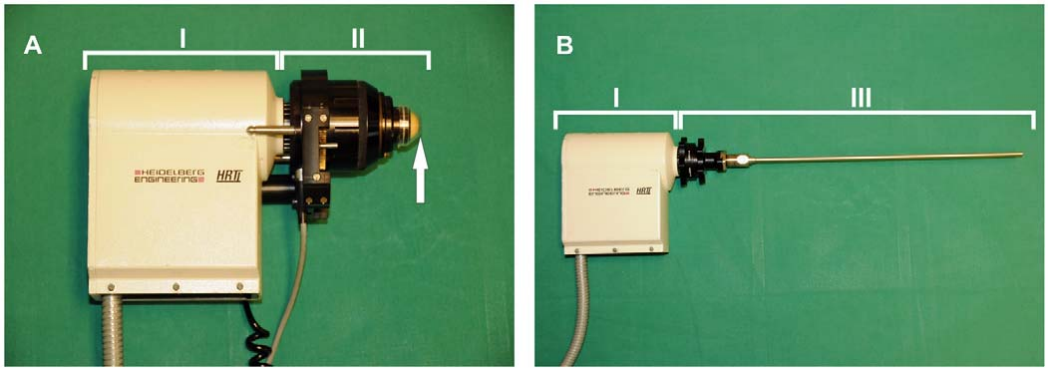
Laser based image modalities. Image modalities 1 (A) und 2 (B) are based on the Heidelberg Retina Tomograph II (I) in combination with the Rostock Cornea Module (II) including the PMMA cover (↑) or the rigid endoscope (III).

Image modality 1 (Figure 1A) is based on the HRT II and the Rostock Cornea Module [13], [18]. The objective lens system comprised a water-immersion objective (Achroplan 63×/0.95 W/AA 1.45 mm, Zeiss, Jena, Germany) and was coupled to the mucosal tissue via a poly (methyl methacrylate) (PMMA) cover to ensure exact depth data by moving the focal plane with a maximum focal shift of 700 µm. The tissue was placed on a gauze cushion that was supported by a foam pad to minimize unintended motion at room temperature. The axial resolution of image modality was determined with 7.6 microns [17].

### Image modality 2

Image modality 2 (Figure 1B) is based on the HRT II and a rigid endoscope (length 23 cm, diameter 5 mm) with an integrated rod lens system (Storz, Tuttlingen, Germany) as described previously [16]. The endoscope coupled to the HRT II attains a spatial resolution of 5 µm in the axial dimension and of 1–2 µm in the lateral dimension. The axial resolution was not yet determined experimentally, but there is evidence of a value of about 12 microns [17].

### Animals

A Mozambique strain of *S. mansoni* maintained in *Biomphalaria glabrata* snails (Brazilian strain) was used throughout this work. Six five-week-old female outbred NMRI mice (Harlan & Winkelman, Netherlands) were infected by soaking in a 50 ml water bath containing 150 cercariae for 90 min as previously described and were euthanised 20 weeks after infection [19]. The experimental protocols were in accordance with the German animal protection law and approved by the regional animal care and use committee (reference number: Landesamt für Landwirtschaft, Lebensmittelsicherheit und Fischerei Mecklenburg Vorpommern, LALLF M-V/TSD/7221.3-2.5.1.002/10).

### Detection of schistosomal eggs within the mucosa of the dissected mouse gut

The large and the small intestine of one mouse were removed, longitudinally sliced and flushed with 0.9% sodium chloride solution to remove faeces and blood. Subsequently, approximately 2 cm long segments of the middle section of the colon and the middle section of the ileum were examined with image modality 1. Visualised eggs were counted and classified for viability.

### Detection of schistosomal eggs within the large intestine by different imaging methods

Five mice were fasted for 12 hours before euthanasia. At autopsy the abdominal cavity was opened and the large intestine and the colon were uncovered. The pelvis was cut in order to ensure penetration of the colon by the rigid endoscope. An approximately 1.5 cm long segment of the colon was examined using image modality 2. Subsequently, this 1.5 cm long colon segment was removed, longitudinally sliced, flushed with 0.9% sodium chloride solution and examined with image modality 1. Finally, a biopsy of 0.5 cm (0.2 cm^2^) diameter was punched out of the middle of the colon area and examined as a crush preparation by bright-field microscopy at 100- to 200-fold magnification. The worm eggs were counted and classified for viability status using all three methods.

### Classification of schistosomal eggs

Based on published classification systems [20]–[21], visualised schistosomal eggs were classified as viable mature, viable immature or dead if at least one of the specific characteristics from Table 1 was observed.

**Table 1:** Characteristics for classification of viable mature, viable immature and dead eggs of *S. mansoni*.

Viable mature eggs	Viable immature eggs	Dead eggs
- fully developed and non-retracted miracidia- movement of the flame cells of the protonephridia or the external cilia- miracidia with twitching movements	- developmental stages 1–4 defined as a non-retracted embryo and various vitelline cells	- retracted , morphologically abnormal miracidia or embryos- eggs with blurry and/or granular contents- eggs without any contents

## Results

### Detection of schistosomal eggs within the mucosa of the dissected mouse gut

**Figure 2:**
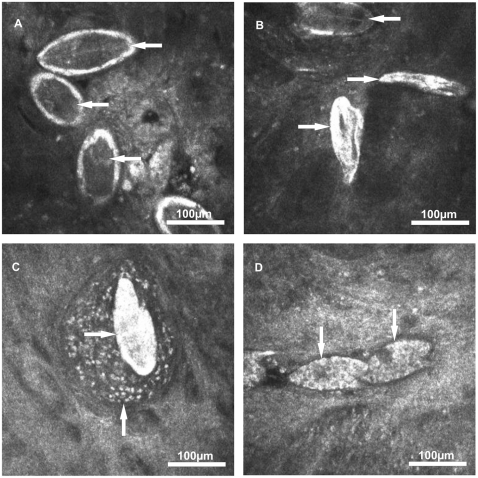
Eggs of *S. mansoni* visualised within the colon mucosa by image modality 1. (A) Viable mature eggs containing dark coloured and fully developed miracidia (←). (B) Dead eggs without any content (→) and a mature egg containing a dark coloured miracidium (←). (C) Dead egg (→) with surrounding granulomatous tissue (↑). (D) Viable immature eggs of developmental stage 1 (↓) containing various vitelline cells.

Within the dissected middle section of the colon 32 *S. mansoni* eggs were detected by scanning the mucosal tissue (Table 2). The eggs firstly appeared as bright structures and after focus adjustment the characteristic egg shape and lateral spine became visible (Video S1). Twenty-eight out of 32 eggs (87.5%) contained dark coloured, fully developed and non-retracted miracidia and were therefore classified as viable mature (Figure 2A). The miracidia of 4 of the 28 mature eggs (14%) showed twitching movements within the eggshell (Video S2). In another 4 out of 32 eggs (12.5%) the eggshells were found to be without any content and were therefore classified as dead (Figure 2B ).

**Table 2:** Total numbers of viable mature, viable immature and dead eggs of *S. mansoni* within the mucosa of the dissected small and large intestine of one mouse examined using image modality 1.

Part of the gut	n _eggs total_	%	n _eggs mature_	%	n _eggs immature_	%	n _eggs dead_	%
Middle section of the colon	32	100	28	88	0	0	4	12
Middle section of the ileum	20	100	14	70	3	15	3	15

Within the dissected middle section of the ileum 20 *S. mansoni* eggs were detected by scanning the mucosal tissue (Table 2). Fourteen out of 20 eggs (70%) were classified as viable mature since they contained dark coloured, fully developed and non-retracted miracidia. Three out of 20 eggs (15%) were classified as viable immature eggs of the developmental stage 1 since they contained various vitelline cells (Figure 2D). Another three out of 20 eggs (15%) were classified as dead. Furthermore, several eggs were found to be surrounded by an egg granuloma (Figure 2C).

### Detection of schistosomal eggs by different imaging methods

After the suitability of CLSM for visualisation and classification of schistosomal eggs within different parts of gut had been evaluated in one mouse, detection of schistosomal eggs was compared by three imaging methods in the colons of five infected mice.

When examining the approximately 1.8±0.4 cm^2^ colon sections with image modality 2, a total of 196 eggs of *S. mansoni* were detected (Table S1). Thirty-six out of 196 eggs (18%) were classified as viable mature (Figure 2A and C), but no miracidia with twitching movements were observed. One-hundred-and-sixty out of 196 eggs (82%) were classified as dead (Figure 2B and C).

**Figure 3:**
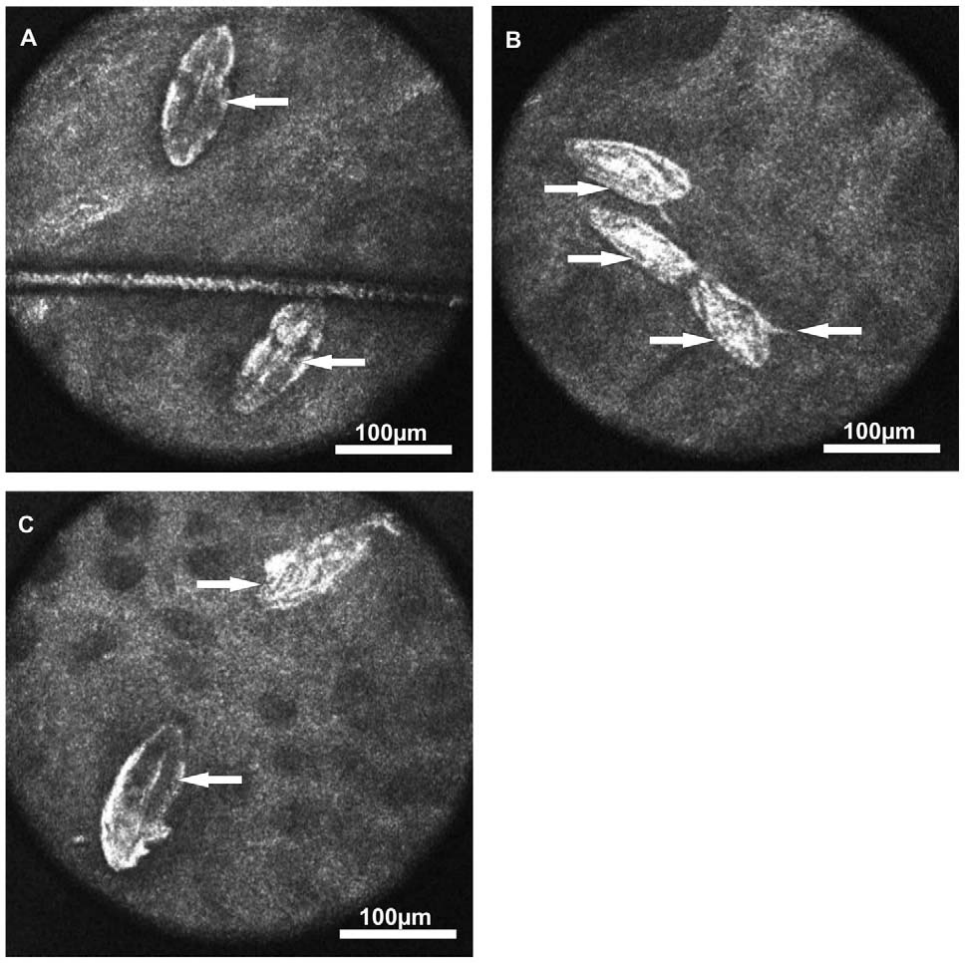
Eggs of *S. mansoni* visualised within the colon mucosa by image modality 2. (A) Viable mature eggs containing dark coloured and fully developed miracidia (←). (B) Dead eggs without any content (→). (C) Dead egg without any content (→) and mature egg containing a dark coloured, fully developed miracidium (←).

When examining the colon sections with image modality 1 a total of 255 eggs were detected (Table S1). Ninety-Seven out of 255 eggs (31%) were classified as viable mature (Figure 3A and C) and the miracidia of three out of 79 mature eggs (4%) showed twitching movements. Six out of 255 eggs (2%) were classified as viable immature eggs of the developmental stage 1. One-hundred-and-seventy out of 255 eggs (67%) were classified as dead (Figure 3B and C).

**Figure 4:**
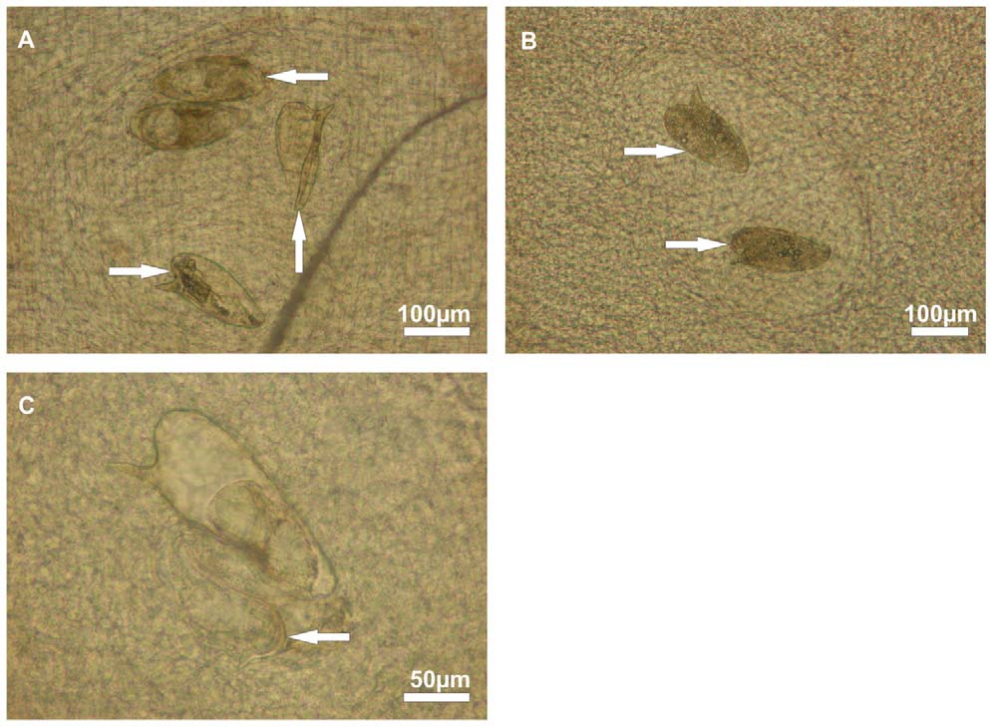
Eggs of *S. mansoni* visualised within a colon biopsy crush preparation by bright-field microscopy. (A) Viable mature eggs containing fully developed miracidia (←), an empty egg shell (↑) of a previously hatched miracidium and a dead egg with diffuse contents (→). (B) Dead eggs without dark and granular contents (→). (C) Egg shell with an almost completely hatched miracidium (←) that is trapped within the tissue.

Within the crush preparations of the 0.2 cm^2^ colon biopsy specimens that were examined by bright-field microscopy a total of 348 eggs were detected (Table S1). Seventy-nine out of 348 eggs (23%) were classified as viable mature (Figure 4A). Eight of the 79 mature eggs (10%) were found to have a twitching miracidium within the eggshell and the miracidium of one egg (1%) was almost hatched into the surrounding tissue (Figure 4C). Another 269 out of 348 eggs (77%) were classified as dead (Figure 4A and B).

## Discussion

This is the first study to demonstrate direct detection of schistosomal eggs within the gut by CLSM. We found that CLSM is an appropriate method for visualising schistosomal eggs within dissected gut tissue and within the intact gut. CLSM permits classification of the viability status of the visualised eggs and differentiates between schistosomal species.

In cases where stool and urine specimens are negative, biopsy specimens from the gut mucosa can be examined for the presence and viability of trapped eggs in tissue crush preparations or in stained paraffin sections. Because biopsies are invasive and the examination area is limited, our study aimed to test the suitability of CLSM for the detection of schistosomal eggs. While focusing through the tissue, viable and dead eggs can be clearly distinguished. Additionally, viable eggs could be further classified into mature and immature, depending on the presence of a miracidium or vitelline cells within the egg shells, and the visualisation of the spine position permits clear differentiation of schistosomal species.

In terms of total egg counts higher numbers of eggs were found during bright-field microscopy of the biopsy crush preparations, since eggs of all layers of the intestine were captured. In contrast, the penetration depth of the laser microscopy is limited and CLSM displays eggs that are located in the mucosal tissue not deeper than 100 µm. However, egg outlines were still visible at depths down to 150 µm; these pictures were blurred and did not permit identification of internal structures or spine position. Image quality is characterised not only by resolution, but also by contrast, depending on illumination level, refraction index and the reflectivity of the tissue studied.

As an indicator of persistent or inactive infection, differentiation between viable and dead eggs is mandatory for therapeutic decisions. The treatment of choice for schistosomiasis is praziquantel. However, since cure rates in some studies are as low as 70% there is an urgent need for a diagnostic tool to monitor treatment success [22]. Miracidia are fully developed approximately one week after egg deposition and subsequently survive in the gut tissue for about 2–5 weeks. Both viable mature and immature eggs indicate the presence of at least one female worm and hence active infection of the host. Thus, the presence of viable eggs approximately 2 months after therapy would indicate treatment failure [23]–[24]. In contrast, the decrease or absence of viable immature eggs after therapy indicates the termination of egg shedding and therefore a possible treatment success [25]–[26].

The examination of biopsy specimens regarding schistosomal eggs can be done by embedding the tissue sample in paraffin followed by the preparation and staining of slides for the microscopic evaluation. Furthermore, biopsy specimens can be examined directly under the bright-field microscope including the miracidium hatching procedure that is only feasible in specialized parasitological laboratories. In comparison to CLSM, where the results are directly available during the examination of the patient, the preparation and evaluation of stained paraffin slides and miracidium hatching assays is time consuming and often of low sensitivity as immature eggs not yet containing miracidia cannot be distinguished from dead eggs [9]. Moreover, invasive biopsies allow the examination of small pieces of the gut mucosa while the non-invasive CLSM allows a larger area to be scanned for eggs.

In conclusion, CLSM visualises schistosomal eggs *in vivo* within the gut, permitting discrimination between schistosomal species and determination of egg viability status. CLSM is therefore a substitute for invasive biopsy procedures and may be an appropriate method for diagnosing intestinal and urinary schistosomiasis in humans during rectoscopy and cystoscopy with respect to the assessment of pathology.

## Supporting Information

Table S1 Total count of viable mature, viable immature and dead schistosomal eggs detected within the mucosa of the colon by different imaging methods.
(DOCX)

Video S1 **Viable eggs of** ***S. mansoni*** **.** The eggs of *S. mansoni* firstly appear as bright structures. After focus adjustment the characteristic lateral spines and the dark coloured miracidia became visible. The eggs were visualized within the colon mucosa by image modality 1.
(AVI)

Video S2 **Twitching movements of a** ***S. mansoni*** **miracidium.** The dark coloured miracidium shows twitching movements within the bright eggshell. The egg was visualized within the colon mucosa by image modality 1.
(AVI)
