# Supplementary material for: Confocal Laser Scanning Microscopy for Detection of Schistosoma mansoni Eggs in the Gut of Mice
Source: PLoS One. 2011 Apr 18;6(4):e18799. doi: 10.1371/journal.pone.0018799 (PMC3078923; doi:10.1371/journal.pone.0018799)
Supplement: Table S1 — Total count of viable mature, viable immature and dead schistosomal eggs detected within the mucosa of the colon by different imaging methods. (DOCX) [file pone.0018799.s001.docx]

| Microscopy system | Image modality 1 | | | | Image modality 2 | | | | Bright-field microscopy | | | |
| --- | --- | --- | --- | --- | --- | --- | --- | --- | --- | --- | --- | --- |
| Examined colon area | 1.8 (±0.4) cm^2^ | | | | 1.8 (±0.4) cm^2^ | | | | 0.2 cm^2^ | | | |
| Egg count | Total | Mature  (%) | Immature  (%) | Dead  (%) | Total | Mature  (%) | Immature  (%) | Dead (%) | Total | Mature  (%) | Immature  (%) | Dead (%) |
| n _eggs mouse 1_ | 55 | 30 (55) | 5 (9) | 20 (36) | 21 | 5 (24) | 0 (0) | 16 (76) | 195 | 55 (28) | 0 (0) | 140 (72) |
| n _eggs mouse 2_ | 20 | 8 (40) | 0 (0) | 12 (60) | 12 | 12 (32) | 0 (0) | 25 (68) | 35 | 3 (19) | 0 (0) | 13 (81) |
| n _eggs mouse 3_ | 78 | 8 (10) | 0 (0) | 70 (90) | 70 | 6 (10) | 0 (0) | 52 (90) | 3 | 6 (8) | 0 (0) | 71 (92) |
| n _eggs mouse 4_ | 86 | 25 (29) | 1 (1) | 60 (70) | 1 | 12 (17) | 0 (0) | 58 (83) | 6 | 9 (26) | 0 (0) | 26 (74) |
| n _eggs mouse 5_ | 16 | 8 (50) | 0 (0) | 8 (50) | 52 | 1 (10) | 0 (0) | 9 (90) | 71 | 6 (24) | 0 (0) | 19 (76) |
| n _eggs mouse 1-5_ | 255 | 79 (31) | 6 (2) | 170 (67) | 196 | 36 (18) | 0 (0) | 160 (82) | 348 | 79 (23) | 0 (0) | 269 (77) |
